# Conventional Pathology Versus Gene Signatures for Assessing Luminal A and B Type Breast Cancers: Results of a Prospective Cohort Study

**DOI:** 10.3390/genes9050261

**Published:** 2018-05-17

**Authors:** Julia E.C. van Steenhoven, Anne Kuijer, Paul J. van Diest, Joost M. van Gorp, Marieke Straver, Sjoerd G. Elias, Jelle Wesseling, Emiel Rutgers, Johanna N.H. Timmer-Bonte, Peter Nieboer, Tineke J. Smilde, Alex Imholz, Charlotte F.J.M. Blanken, Sabine Siesling, Thijs van Dalen

**Affiliations:** 1 Department of Surgery, Diakonessenhuis Utrecht, 3582 KE Utrecht, The Netherlands; akuijer@diakhuis.nl (A.K.); tvdalen@diakhuis.nl (T.v.D.); 2 Department of Pathology, University Medical Center Utrecht, 3584 CX Utrecht, The Netherlands; P.J.vanDiest@umcutrecht.nl; 3 Department of Pathology, Diakonessenhuis Utrecht, 3582 KE Utrecht, The Netherlands; jvgorp@diakhuis.nl; 4 Department of Surgery, Haaglanden Medical Center, 2512 VA The Hague, The Netherlands; mstraver@hotmail.com; 5 Department of Epidemiology, Julius Center for Health Sciences and Primary Care, University Medical Center Utrecht, Utrecht University, 3584 CG Utrecht, The Netherlands; s.elias@umcutrecht.nl; 6 Department of Pathology, Netherlands Cancer Institute—Antoni van Leeuwenhoek Hospital, 1066 CX Amsterdam, The Netherlands; j.wesseling@nki.nl; 7 Department of Surgery, Netherlands Cancer Institute—Antoni van Leeuwenhoek Hospital, 1066 CX Amsterdam, The Netherlands; erutgers@nki.nl; 8 Department of Medical Oncology, Alexander-Monro Hospital, 3723 MB Bilthoven, The Netherlands; anja.timmer-bonte@radboudumc.nl; 9 Department of Medical Oncology, Radboud University Medical Center, 6525 GA Nijmegen, The Netherlands; 10 Department of Medical Oncology, Wilhelmina Hospital Assen, 9401 RK Assen, The Netherlands; peter.nieboer@wza.nl; 11 Department of Medical Oncology, Jeroen Bosch Hospital, 5223 GZ 's-Hertogenbosch, The Netherlands; T.smilde@jbz.nl; 12 Department of Medical Oncology, Deventer Hospital, 7416 SE Deventer, The Netherlands; A.imholz@dz.nl; 13 Department of Surgery, Rijnstate Hospital, 6815 AD Arnhem, The Netherlands; cblankenpeeters@gmail.com; 14 Department of Research, Netherlands Comprehensive Cancer Organization, 3511 DT Utrecht, The Netherlands; s.siesling@iknl.nl; 15 Department of Health Technology and Services Research, MIRA Institute for Biomedical Technology and Technical Medicine, University of Twente, 7522 NB Enschede, The Netherlands

**Keywords:** breast cancer, molecular subtyping, local pathology, 80-gene signature, 70-gene signature, Ki67

## Abstract

In this study, in estrogen receptor positive (ER+) early stage breast cancer patients who were considered candidates for 70-gene signature (70-GS, “MammaPrint”) use, we compared molecular subtyping (MS) based on the previously validated 80-gene signature (80-GS, “BluePrint”) versus surrogate pathological subtyping (PS). Between 1 January 2013 and 31 December 2015, 595 clinical intermediate risk ER+ early stage breast cancer patients were enrolled. Hormone receptor (HR) and HER2 receptor status were determined by conventional pathology using immunohistochemistry (IHC) and fluorescent in situ hybridization (FISH). Ki67 was assessed in a subset of patients. The overall concordance between PS and MS for luminal type cancers (A and B together) was 98%. The concordance between PS and MS for luminal A and luminal B type cancers based on the Bloom Richardson histological grade (BR) (*n* = 586) or Ki67 (*n* = 185) was low: 64% (Kappa 0.20 [95% CI 0.11–0.28]) and 65% (Kappa 0.22 [95% CI 0.062–0.37]), respectively. In this prospective study (NCT02209857) of a selection of ER+ and predominantly HER2− early-stage breast cancer patients, the additional ability of the 80-GS to distinguish between luminal, HER2-type and basal-like cancers was inherently very limited. The distinction of luminal-type tumors into A and B according to Ki67 status or BR grade versus the 70-GS revealed poor concordance.

## 1. Introduction 

The identification of intrinsic (“molecular”) subtypes of human breast cancer tumors by Perou et al. 15 years ago catalyzed the concept of individualized cancer therapy [1]. The improved understanding of molecular subtypes is of clinical importance, as different subtypes require specific treatment regimens and are associated with different outcomes. The majority of early stage breast cancer patients are diagnosed with an estrogen receptor positive (ER+) and HER2 receptor negative (HER2−) disease, associated with favorable outcomes, and patients benefit from endocrine therapy. HER2-driven and basal-like tumors are more aggressive breast cancer subtypes, and patients are sensitive to chemotherapy. The population diagnosed with ER+/HER2− (luminal type) disease is highly heterogeneous as patients with similar clinicopathological features can have strikingly different outcomes. In patients diagnosed with luminal A type disease, the additional value of chemotherapy over endocrine is questionable, whereas chemotherapy seems to be of more benefit to patients with luminal B tumors [2,3,4].

In clinical practice, pathological determination of the estrogen receptor (ER), progesterone receptor (PR), HER2-status (HER2), Bloom Richardson histological grade (BR) and Ki67 are generally used to determine surrogate intrinsic cancer subtypes [5]. Gene expression profiles may be used in patients with ER+ breast cancer to make a distinction between groups with a low or high risk of developing distant metastases in order to optimize patient selection for chemotherapy [6,7,8,9,10,11,12]. Based on the expression of 80-genes, a molecular subtyping profile (“BluePrint”) has been developed for the stratification of breast cancer tumors into the three main molecular subtypes: luminal, HER2 and basal [13]. By adding the prognostic risk profile of the 70-gene signature (70-GS), a substratification of luminal-type tumors into low risk (luminal A) and high risk (luminal B) type cancers can be made [14].

The St. Gallen International Expert Consensus panel defined a surrogate to distinguish luminal A type breast cancer from luminal B-type, based on a combination of ER, PR, HER2 and the expression of the proliferation marker, Ki67 [15,16]. Furthermore, histological grade is also being used as an alternative to Ki67 in luminal-type breast cancers. While hormone and HER2 receptor statuses have been proven to be highly reproducible [17], a standardized methodology for Ki67 level assessment is lacking, and the role of Ki67 in clinical decision-making remains uncertain [18,19,20,21,22]. Reliable distinction of luminal-type tumors into A and B is important, since the therapeutic consequences of this are large. Misinterpretation of these surrogates may lead to the risk of patients being over- or under-treated.

As part of a prospective observational multicenter study in a selection of ER+ breast cancer patients who were considered candidates for 70-GS use [23], a conventional pathology assessment was performed as well as gene expression profiling. We assessed molecular subtypes (luminal/HER2/basal) using an 80-gene signature (80-GS) in ER+, mostly HER2− and partly HER2+ cancers determined by conventional pathology. In addition, concordance between luminal A and B-type cancers was evaluated using local pathology, stratified by Ki67 status or BR grade versus gene signatures (80-GS/70-GS).

## 2. Materials and Methods

### 2.1. Patients

As part of a prospective observational multicenter study regarding the influence of the 70-GS on adjuvant chemotherapy decision-making in patients with surgically-treated ER+ breast cancer, conventional (local) pathology data and gene expression read-outs were obtained between 1 January 2013 and 31 December 2015. The study was approved by the medical ethics committee of the University Medical Center Utrecht (12-450) and by institutional review boards of participating centers. The study protocol (protocol number 12-450) was registered in the clinicaltrial.gov database (NCT02209857). Within the study, patients diagnosed with early stage ER+ invasive ductal breast cancer with an uncertain benefit of adjuvant chemotherapy based on traditional prognostic factors were eligible for inclusion. Twenty-three out of thirty-three participating hospitals offered patients the opportunity for their tumor samples to be additionally evaluated by the 80-GS (BluePrint ©). In total, 595 patients treated in these 23 hospitals had both tests performed, and these patients were included in the present study.

### 2.2. Routine Pathology Assessment 

The determination of hormone receptor (HR) status (ER and PR) and HER2-receptor status was done routinely and locally in the pathology labs in accordance with national pathology guidelines. ER and PR status were routinely determined by immunohistochemistry (IHC) and positive identification was defined as the presence of nuclear staining in ≥10% of breast cancer cells, in accordance with the Dutch Breast Cancer Guidelines [24]. The results were identical if we applied a cut-off of ≥1%, as all cases included in this study with any ER or PR positive staining of the nuclei, showed at least 10% positive nuclear staining [25].

HER2 expression was scored by IHC in accordance with international guideline recommendations [26]: 0 if no staining was observed or membrane staining was incomplete and faint/barely perceptible and within less than 10% of the tumor cells, 1+ if staining was incomplete and faint/barely perceptible, but within more than 10% of the tumor cells, 2+ if more than 10% of the tumor cells displayed circumferential staining of moderate intensity or complete and circumferential strong staining within less than 10% of the tumor cells, and 3+ for strong circumferential membrane staining within ≥10% of the tumor cells. A tumor was considered HER2-negative when a score of 0 or 1+ was found and positive when a score of 3+ was observed. Tumors with 2+ HER2 expression were additionally evaluated by HER2 fluorescent in situ hybridization (FISH). In accordance with the Dutch guidelines, the cut-offs for HER2 low level and high-level amplification were defined as >6 and >10 copies of the HER2 gene or clusters, respectively [23]. Ki67 assessment was routinely performed in 11 of the participating hospitals, and the tests were done in five different pathology laboratories. When Ki67 had been determined (*n* = 185), the average scoring method was performed and a Ki67 cut-off value of 20% [25] was used for the designation of Ki67 into luminal A or luminal B type tumors.

### 2.3. Pathological Subtyping (PS)

In accordance with the 2013 recommendations from the St. Gallen guidelines, surrogate molecular subtypes were determined as follows: luminal A-like (ER+ and PR ≥20%, HER2− and Ki67 <20%) and luminal B-like (ER+/HER2−/PR <20%, or ER+/HER2−/Ki67 ≥20%, or ER+/HER2+) [22]. Using surrogate molecular subtyping based on grade, Bloom Richardson (BR) histological 1 and 2 were combined into the low proliferative group and BR grade 3 represented the high proliferative tumors, and surrogate subtypes were determined as follows: luminal A- like (ER+ and PR ≥20%, HER2− and BR I/II) and luminal B-like (ER+/HER2−/PR <20%, or ER+/HER2−/BR III or ER+/HER2+). 

### 2.4. Molecular Subtyping (MS)

All tumor samples were routinely evaluated by the 80-GS and 70-GS methods at the Agendia Laboratory in Amsterdam, The Netherlands; the individuals who conducted the analysis were blinded to clinical and pathological data. The 80-GS (“BluePrint”) stratified breast cancers into the molecular subtypes: luminal, HER2, and basal-like [13]. Combining the 80-GS with the 70-GS method enabled further stratification of luminal tumors into luminal A (70-GS low risk) and luminal B (70-GS high risk) [14].

### 2.5. Statistical Analysis

A comparison of molecular subtyping (MS) and pathological subtyping (PS) was done with two by three (Table 2) and two by four (Table 3) contingency tables, calculating overall concordance. In addition, comparison of pathological subtyping (PS) and molecular subtyping (MS) for Luminal A and Luminal B tumors based on BR grade and Ki67 was done with the Kappa statistic to evaluate the agreement between these two classifications on a nominal scale and accompanying 95% confidence intervals (CI) were calculated [27]. 

## 3. Results

### 3.1. Patients

There were 595 patients with a median age of 58 years. The majority of the patients had intermediate grade tumors (74%), with no or micro-metastasic axillary lymph node involvement (pN0 or pN1mi ≥93%, Table 1). A local pathology assessment determined that all 595 patients had ER+ tumors, 87% had PR+ and 2% had HER2+ tumors. The 70-GS classified 59% of the patients as having a ‘low risk’ form of breast cancer. In the subset of 185 patients in whom Ki67 levels were assessed, 83% had Ki67 levels <20%, reflecting a low risk. This subset of patients had comparable proportions of PR+ and HER2+ tumors (88% and 1%, respectively) (Table 1).

### 3.2. Pathological Subtyping Versus Molecular Subtyping Using the 80-GS Only 

Using local pathology, 98% of patients (*n* = 576) were regarded as [HR+/HER2−, luminal A] and 2% (*n* = 12) of patients as [HR+/HER2+, luminal B]. In seven patients (1%), the HER2 receptor status was not conclusive and these individuals were therefore excluded from the analysis. The 80-GS classified 98% (*n* = 583) of all patients as luminal-type, 1% (*n* = 7) as HER2-type and 1% (*n* = 5) as basal-type. The comparison of MS and PS for luminal A and B together resulted in an overall concordance of 98% (Table 2). The ER expression percentages of patients reclassified as HER2-type were 50% (*n* = 1), 70% (*n* = 1), 90% (*n* = 1) or 100% (*n* = 4). The ER expression percentages of patients reclassified as basal-type were 10% (*n* = 1), 50% (*n* = 2), 70% (*n* = 1) or 100% (*n* = 1).

### 3.3. Comparison of Luminal A and Luminal B tumors by Molecular or Pathological Subtyping 

Based on the BR grade, stratification of luminal-type tumors into A and B was performed. Using PS, 74% of patients (*n* = 448) were classified as luminal A, and 26% of patients (*n* = 138) were classified as luminal B. Using MS, 58% of patients (*n* = 342) were classified as luminal A, 40% of patients (*n* = 232) as luminal B, 1% of patients (*n* = 7) as HER2-type and 1% of patients (*n* = 5) as basal-type. Thirty-four percent of patients (*n* = 154) considered to be PS luminal A were reclassified as MS luminal B. Thirty-eight percent of patients (*n* = 52) regarded as PS luminal B were reclassified as MS luminal A. The overall concordance between PS and MS was 63%. The concordance between PS and MS within the luminal group was 64% (Kappa 0.20 [95% CI 0.11–0.28], Table 3). 

### 3.4. Comparison of Luminal A and Luminal B tumors by Molecular and Pathological Subtyping

Based on hormone and HER2 receptors and Ki-67 status, stratification of molecular subtypes into luminal A or luminal B tumors could be performed. Based on local pathology, 82% (*n* = 151) were classified as PS luminal A and 18% (*n* = 34) as PS luminal B. Of the patients classified as PS Luminal A, 64% (*n* = 96) were also classified as MS luminal A. Thirty-four percent of patients (*n* = 52) and 2% (*n* = 3) of patients were reclassified as MS luminal B and MS HER2-type, respectively. Of the patients classified as PS luminal B type, 22% (*n* = 65) were also classified as MS luminal B. Eleven percent of patients (*n* = 32) and 1% of patients (*n* = 3) were reclassified as MS luminal A type and MS basal-type, respectively. The overall concordance between PS and MS in this subset of patients was 64%. The concordance between PS and MS within the luminal group was 65% (Kappa 0.22 [95% CI 0.062–0.37]) (Table 4).

## 4. Discussion

In this prospective multicenter ER+ breast cancer study, we observed high concordance between conventional pathology assessment and gene-expression profiling for luminal-type cancers (A and B together). When Ki67 expression or BR grade was used in addition to routine pathology and compared to molecular subtyping to differentiate between luminal A/B type cancers, concordance was low.

The current study, therefore, shows that molecular subtyping using local pathology or 80-GS results in classification of similar proportions of luminal-type tumors. The observed concordance is in line with a previous study conducted by Nguyen et al. (*n* = 135) in which concordance between luminal-type tumors by IHC/FISH and 80-GS was 96% [28]. Similarly, results of a study evaluating the effect of locally and centrally assessed hormone and HER2 receptor statuses revealed comparable proportions of tumors classified as ER+ and HER2−97% and 98%, respectively. In the latter study, central reclassification rates were higher in tumors originally assessed as ER− (14%) and HER2+ (21%). These results suggest that 80-GS based molecular subtyping has little additional value in patients that are classified as HR+/HER2− by conventional pathology. 

In the present study, stratification of luminal-type tumors into types A and B by conventional pathology based on the expression of the proliferation marker, Ki67, compared to 70-GS was associated with lower concordance (64%). This apparent discordance within the luminal group is in line with results reported in the MINDACT trial where 54% of patients with a luminal B subtype according to conventional pathology were reclassified as luminal A by the 70/80-GS [29]. As a consequence, using conventional or microarray analysis (70/80-GS) for breast cancer subtyping may lead to discordant chemotherapy decision-making with the risk of patients being potentially over or undertreated.

The pathological distinction between ER+ tumors into low risk (luminal A) and high risk (luminal B) for developing metastases is mostly based on the assessment of proliferative activity. Histologic grade, in which the mitotic activity index as measure of proliferation plays a major role, is generally used to this end, but the St. Gallen breast cancer consensus panel also recommends Ki67 as a means of determining proliferative activity and therefore, selecting patients for chemotherapy. However, a standardized methodology for Ki67 assessment is lacking [18] and revisions within the St. Gallen expert panel for the most appropriate cut-off for high proliferative tumors are still pending [30,31,32,33]. In accordance with the 2013 St Gallen recommendations, we set the Ki67 cut-off for high proliferation at 20%. If a 14% Ki67 cut-off value had been applied, the comparison between low risk and high risk tumors would have resulted in even higher discordance (37%) of tumors reclassified by the 80- and 70-GS methods (Supplementary Materials Table S1). Intra-tumoral heterogeneity of Ki67 expression levels, inter-laboratory and inter-observer variability of Ki67 staining and differences in Ki67 Labelling Index values have been observed by others and hamper the utility of this biomarker as a reproducible prognostic tool [18,22,34,35]. In our study, 11 hospitals and five different pathology labs were used to determine Ki67. Among these pathology labs, different staining methods may exist and could have caused the discordance between molecular and pathological subtyping.

In this prospective multicenter trial, molecular subtyping by local pathology versus gene signatures was evaluated for a large group of patients. Furthermore, this selection of ER+ patients is very relevant as this is the subset of patients in whom gene signatures are most commonly deployed to guide chemotherapy decisions and, as such, best reflects current clinical practice. Unfortunately, the composition of our study population precluded the performance of 80-GS in HER2 driven and basal-like cancers and so, to be able to study these groups, future studies need to be performed.

It is noteworthy that discordance between conventional pathology and the 80-GS for HER2-driven tumor types reported in trials in neo-adjuvant setting, is high [4,36,37]. In the Neoadjuvant Breast Register Symphony Trial (NBRST), approximately half of patients (48%) regarded as HER2+/ER+ by conventional pathology were classified as BluePrint luminal-type. In addition, a comparison of molecular subtyping in clinical HER2+ patients in the MINDACT trial revealed 38% and 5% of patients reassigned by the BluePrint as luminal-type and basal-type, respectively. These results confirm the presence of different underlying dominant pathways indicating that expression of the luminal pathway is often dominant compared to the HER2-driven tumor profile.

In conclusion, in this prospective study of a selection of ER+ and predominantly HER2− early-stage breast cancer patients, the additional value of the 80-GS to distinguish between luminal, HER2-type and basal like cancers was inherently very limited. However, agreement between luminal A and luminal B type tumors based on local pathology enhanced by Ki-67 or BR grade versus the 70-GS, was poor. The main implication of our study is the existence of disparity between the two classification methods and the concomitant risk of inadequate treatment allocation. In that regard, there may be a role for gene expression profiling as a consistent tool to discriminate between luminal A and B to guide adjuvant chemotherapy decision-making.

## Figures and Tables

**Table 1:** Baseline characteristics in patients assessed by local pathology (*n* = 595) and in patients assessed by local pathology enhanced by Ki67 level determination (*n* = 185).

**Characteristics**	**Total *n* = 595 (%, valid)**	**Subset Ki67 *n* = 185 (%, valid)**
**Age, years, median**	58	57
**(*range*)**	(35–80)	(35–74)
**Pathological T-stage**		
T1	480 (80.6)	153 (82.7)
T2	114 (19.2)	31 (16.8)
T3	1 (0.2)	1 (0.5)
**Pathological N-stage**		
N0(i+)	496 (84.5)	164 (89.6)
N1mi	54 (9.2)	11 (6)
N1(a-c)	37 (6.3)	8 (4.4)
Nx	8	2
**Tumor grade**		
1	86 (14.5)	30 (16.3)
2	438 (73.7)	125 (67.9)
3	70 (11.8)	29 (15.8)
Unknown	1	1
**ER status**		
ER+	595 (100)	185 (100)
**PR status**		
PR+	518 (87.2)	163 (88.6)
PR-	76 (12.8)	21 (11.4)
Unknown	1	1
**HER2 status**		
HER2+	12 (2)	2 (1.1)
HER2−	576 (98)	182 (98.9)
Unknown	7	1
**Ki67 Level**		
<20%, low	153 (83)	153 (83)
≥20%, high	32 (17)	32 (17)
Not assessed	410	-
**70-GS**		
Low risk	349 (59)	109 (59)
High risk	246 (41)	76 (41)

ER: estrogen receptor; PR: progesterone receptor; HER2: human epidermal growth receptor 2; Ki-67: proliferation marker; N0/N0(i+): no axillary lymph node involvement/isolated tumor cells; Nmi: micro-metastasis; N1a-c: metastasis in movable ipsilateral. Level I, II axillary lymph node(s), Nx axillary lymph node status not assessed.

**Table 2:** Pathological subtyping using hormone and HER2 receptor status versus molecular subtyping using the 80-GS (*n* = 588).

**Molecular Subtypes**				
**Clinical Subtypes**	**80-GS Luminal (%)**	**80-GS HER2 (%)**	**80-GS Basal (%)**	**Total**
ER+/PR+, HER2−	567 (98)	4 (1)	5 (1)	576
ER+/PR+, HER2+	9 (75)	3 (25)	0 (0)	12
Total	576	7	5	588

80-GS: 80-gene signature; ER: estrogen receptor; PR: progesterone receptor; HER2: human epidermal growth factor receptor 2. The overall concordance between PS and MS was 98%.

**Table 3:** Comparison of pathological subtyping using Bloom Richardson histological grade versus molecular subtyping (*n* = 586).

**Molecular Subtypes**					
**Clinical Subtypes**	**Luminal A (%)**	**Luminal B (%)**	**HER2 (%)**	**Basal (%)**	**Total**
ER+, PR ≥ 20%, HER2−, BR I/II	290 (65)	154 (34)	4 (1)	-	448
ER+ & (PR < 20%, or HER2+ or BR III)	52 (38)	78 (57)	3 (2)	5 (3)	138
Total	342	232	7	5	586

GS: 70-gene signature; 80-GS: 80-gene signature; ER: estrogen receptor; PR: progesterone receptor; HER2: human epidermal growth factor receptor 2. The overall concordance between PS and MS was 63%. The concordance between PS and MS within the luminal group was 64% (Kappa 0.20 [95% CI 0.11–0.28]).

**Table 4:** Comparison of pathological subtyping based on Ki67 status versus molecular subtyping (*n* = 185).

**Molecular Subtypes**					
**Clinical Subtypes**	**Luminal A (%)**	**Luminal B (%)**	**HER2 (%)**	**Basal (%)**	**Total**
ER+, PR ≥ 20%, HER2−, Ki67 < 20%	96 (64)	52 (34)	3 (2)	0	151
ER+ & (PR < 20%, or HER2+ or Ki67 ≥ 20%)	11 (32)	22 (65)	0	1 (3)	34
Total	107	74	3	1	185

70-GS: 70-gene signature; 80-GS: 80-gene signature; ER: estrogen receptor; PR: progesterone receptor; HER2: human epidermal growth factor receptor 2; Ki-67: proliferation marker protein. The overall concordance between PS and MS was 64%. The concordance between PS and MS within the luminal group was 65% (Kappa 0.22 [95% CI 0.062–0.37]).

## Supplementary Materials

The following are available online at http://www.mdpi.com/2073-4425/9/5/261/s1, Table S1: Comparison of Luminal A and Luminal B type tumors with Ki67 versus gene-signatures. The datasets used and/or analyzed during the current study are available from the corresponding author on reasonable request.

## References

[1] Perou C.M., Sørlie T., Eisen M.B., van de Rijn M., Jeffrey S.S., Rees C.A., Pollack J.R., Ross D.T., Johnsen H., Akslen L.A. (2000). Molecular portraits of human breast tumours. Nature.

[2] Sorlie T., Perou C.M., Tibshirani R., Aas T., Geisler S., Johnson H., Hastie T., Eisen M.B., van de Rijn M., Jeffrey S.S. (2001). Gene expression patterns of breast carcinomas distinguish tumor subclasses with clinical implications. Proc. Natl. Acad. Sci. USA.

[3] Glück S., de Snoo F., Peeters J., Stork-Sloots L., Somlo G. (2013). Molecular subtyping of early-stage breast cancer identifies a group of patients who do not benefit from neo-adjuvant chemotherapy. Breast Cancer Res. Treat..

[4] Whitworth P., Stork-Slooks L., de Snoo F.A., Richards P., Rotkis M., Beatty J., Mislowsky A., Pellicane J.V., Nguyen B., Lee L. (2014). Chemosensitivity predicted by BluePrint 80-Gene functional subtype and MammaPrint in the Prospective Neoadjuvant Breast Registry Symphony Trial (NBRST). Ann. Surg. Oncol..

[5] Tang P., Tse G.M. (2016). Immunohistochemical surrogates for molecular classification of breast carcinoma: A 2015 update. Arch. Pathol. Lab. Med..

[6] Van’t Veer L.J., Dai H., van de Vijver M.J., He Y.D., Hart A.A., Mao M., Peterse H.L., van der Kooy K., Marton M.J., Witteveen A.T. (2002). Gene expression profiling predicts clinical outcome of breast cancer. Nature.

[7] Van de Vijver M.J., He Y.D., van’t Veer L.J., Dai H., Hart A.A., Voskuil D.W., Schreiber G.J., Peterse J.L., Roberts C., Marton M.J. (2002). A gene-expression signature as a predictor of survival in breast cancer. N. Engl. J. Med..

[8] Cardoso F., van’t Veer L.J., Bogaerts J., Slaets L., Viale G., Delaloge S., Pierga J.Y., Brain E., Causeret S., DeLorenzi M. (2016). 70-gene signature as an aid to treatment decisions in early-stage breast cancer. N. Engl. J. Med..

[9] Paik S., Shak S., Tang G., Kim C., Baker J., Cronin M., Baehner F.L., Walker M.G., Watson D., Park T. (2004). A multigene assay to predict recurrence of tamoxifen-treated, node-negative breast cancer. N. Engl. J. Med..

[10] Nielsen T.O., Parker J.S., Leung S., Voduc D., Ebbert M., Vickery T., Davies S.R., Snider J., Stijleman I.J., Reed J. (2010). A comparison of PAM50 intrinsic subtyping with immunohistochemistry and clinical prognostic factors in tamoxifen-treated estrogen receptor–positive breast cancer. Clin. Cancer Res..

[11] Parker J.S., Mullins M., Cheang M.C., Leung S., Voduc D., Vickery T., Davies S., Fauron C., He X., Hu Z. (2009). Supervised risk predictor of breast cancer based on intrinsic subtypes. J. Clin. Oncol..

[12] Filipits M., Rudas M., Jakesz R., Dubsky P., Fitzal F., Singer C.F., Dietze O., Greil R., Jelen A., Sevelda P. (2011). A new molecular predictor of distant recurrence in ER-positive, HER2−negative breast cancer adds independent information to conventional clinical risk factors. Clin. Cancer Res..

[13] Krijgsman O., Roepman P., Zwart W., Carroll J.S., Tian S., de Snoo F.A., Bender R.A., Bernards R., Glas A.M. (2012). A diagnostic gene profile for molecular subtyping of breast cancer associated with treatment response. Breast Cancer Res. Treat..

[14] Yao K., Goldschmidt R., Turk M., Wesseling J., Stork-Sloots L., de Snoo F., Cristofanilli M. (2015). Molecular subtyping improves diagnostic stratification of patients with primary breast cancer into prognostically defined risk groups. Breast Cancer Res. Treat..

[15] Scholzen T., Gerdes J. (2000). The Ki-67 protein: From the known and the unknown. J. Cell. Physiol..

[16] Prat A., Cheang M.C., Martín M., Parker J.S., Carrasco E., Caballero R., Tyldesley S., Gelmon K., Bernard P.S., Nielsen T.O. (2013). Prognostic significance of progesterone receptor-positive tumor cells within immunohistochemically defined luminal A breast cancer. J. Clin. Oncol..

[17] Ekholm M., Grabau D., Bendahl P.O., Bergh J., Elmberger G., Olsson H., Russo L., Viale G., Fernö M. (2015). Highly reproducible results of breast cancer biomarkers when analyzed in accordance with national guidelines—A Swedish survey with central re-assesment. Acta Oncol..

[18] Focke C.M., van Diest P.J., Decker T. (2016). St Gallen 2015 subtyping of luminal breast cancers: Impact of different Ki67-based proliferation assessment methods. Breast Cancer Res. Treat..

[19] Cheang M.C., Chia S.K., Voduc D., Gao D., Leung S., Snider J., Watson M., Davies S., Bernard P.S., Parker J.S. (2009). Ki67 index, HER2 status, and prognosis of patients with luminal B breast cancer. J. Natl. Cancer Inst..

[20] Dowsett M., Nielsen T., A’Hern R., Bartlett J., Coombes R.C., Cuzick J., Ellis M., Henry N.L., Hugh J.C., Lively T. (2011). Assessment of Ki67 in breast cancer: Recommendations from the International Ki67 in Breast Cancer working group. J. Natl. Cancer Inst..

[21] Bueno-de-Mesquita J.M., Nuyten D.S., Wesseling J., van Tinteren H., Linn S.C., van de Vijver M.J. (2010). The impact of inter-observer variation in pathological assessment of node-negative breast cancer on clinical risk assessment and patient selection for adjuvant systemic treatment. Ann. Oncol..

[22] Varga Z., Cassoly E., Li Q., Oehlschlegel C., Tapia C., Lehr H.A., Klingbiel D., Thürlimann B., Ruhstaller T. (2015). Standardization for Ki-67 Assessment in Moderately Differentiated Breast Cancer. A Retrospective Analysis of the SAKK 28/12 Study. PLoS ONE.

[23] Kuijer A., Straver M., Dekker B.D., van Bommel A.C.M., Elias S.G., Smorenburg C.H., Wesseling J., Linn S.C., Rutgers E.J.Th, Siesling S. (2017). Impact of 70-gene signature use on adjuvant chemotherapy decisions in patients with estrogen receptor-positive early stage breast cancer: Results of a prospective cohort study. J. Clin. Oncol..

[24] Kwaliteitsinstituut voor de Gezondheidszorg CBO en Vereniging van Integrale Kankercentra (2012). Pathologie, Richtlijn mammacarcinoom.

[25] Hammond M.E.H., Hayes D.F., Dowsett M., Allred D.C., Hagerty K.L., Badve S., Fitzgibbons P.L., Francis G., Goldstein N.S., Hayes M. (2010). American society of clinical oncology/college of American pathologists guideline recommendations for immunohistochemical testing of estrogen and progesterone receptors in breast cancer. J. Clin. Oncol..

[26] Wolff A.C., Hammond M.E., Hicks D.G., Dowsett M., McShane L.M., Allison K.H., Allred D.C., Bartlett J.M., Bilous M., Fitzgibbons P. (2013). Recommendations for human epidermal growth factor receptor 2 testing in breast cancer: American Society of Clinical Oncology/College of American Pathologists clinical practice guideline update. J. Clin. Oncol..

[27] (2016). Rstudio.

[28] Nguyen B., Cusomano P.G., Deck K., Kerlin D., Garcia A.A., Barone J.L., Rivera E., Yao K., de Snoo F.A., van den Akker J. (2012). Comparison of Molecular subtyping with BluePrint, MammaPrint, and TargetPrint to local clinical subtyping in breast cancer patients. Ann. Surg. Oncol..

[29] Viale G., de Snoo F., Slaets L., Bogaerts J., van’t Veer L., Rutgers E.J., Piccart-Gebhart M.J., Stork-Sloots L., Glas A., Russo L. (2017). Immunohistochemical versus molecular (BluePrint and MammaPrint) subtyping of breast carcinoma. Outcome results from the EORTC 10041/BIG 3-04 MINDACT trial. Breast Cancer Res. Treat..

[30] Goldhirsch A., Winer E.P., Coates A.S., Gelber R.D., Piccart-Gebhart M., Thürlimann B., Senn H.J. (2013). Personalizing the treatment of woman with early breast cancer: Highlights of the St. Gallen International Expert consensus on the Primary Therapy of Early Breast Cancer 2013. Ann. Oncol..

[31] Goldhirsch A., Ingle J.N., Gelber R.D., Coates A.S., Thürlimann B., Senn H.J. (2009). Thresholds for therapies: Highlights of the St. Gallen International Expert Consensus on the primary therapy of early breast cancer, 2009. Ann. Oncol..

[32] Goldhirsch A., Wood W.C., Coates A.S., Gelber R.D., Thürlimann B., Senn H.J. (2011). Strategies for subtypes—dealing with the diversity of breast cancer: Highlights of the St. Gallen International Expert Consensus on the Primary Therapy of Early Breast Cancer 2011. Ann. Oncol..

[33] Coates A.S., Winer E.P., Goldhirsch A., Gelber R.D., Gnant M., Piccart-Gebhart M., Thürlimann B., Senn H.J. (2015). Tailoring therapies—Improving the management of early breast cancer: St. Gallen international Expert consensus on the Primary Therapy or Early Breast Cancer 2015. Ann. Oncol..

[34] Urriticoechea A., Smith I.E., Dowsett M. (2005). Proliferation marker Ki-67 in early stage breast cancer. J. Clin. Oncol..

[35] Rakha E.A., Ellis I.O. (2007). An overview of assessment of prognostic and predictive factors in breast cancer needle core biopsy specimens. J. Clin. Pathol..

[36] Whitworth P., Beitsch P., Mislowsky A., Pellicane J.V., Nash C., Murray M., Lee L.A., Dul C.L., Rotkis M., Baron P. (2017). Chemosensitivity and endocrine sensitivity in clinical luminal breast cancer patient in the Prospective Neoadjuvant Breast Registry Symphony Trial (NBRST) predicted by molecular subtyping. Ann. Surg. Oncol..

[37] Beitsch P., Whitworth P., Baron P., Rotkis M.C., Mislowsky A.M., Richards P.D., Murray M.K., Pellicane J.V., Dul C.L., Nash C.H. (2017). Pertuzumab/Trastuzumab/CT versus Trastuzumab/CT therapy for HER2+ breast cancer: Results from the Prospective Neoadjuvant Breast Registry Symphony Trial (NBRST). Ann. Surg. Oncol..

