# Supplementary material for: Conventional Pathology Versus Gene Signatures for Assessing Luminal A and B Type Breast Cancers: Results of a Prospective Cohort Study
Source: Genes (Basel). 2018 May 17;9(5):261. doi: 10.3390/genes9050261 (PMC5977201; doi:10.3390/genes9050261)
Supplement: Supplementary file 1 [file genes-09-00261-s001.pdf]

Supplementary table 1 Comparison of Luminal A and Luminal B type tumors with Ki67 versus gene-signatures (70-GS/80-GS) (n=179)

	Molecular subtypes		
**Clinical subtypes**	**Luminal A (%)**	**Luminal B (%)**	**Total**
**ER+, PR ≥20%, HER2-, Ki67 <14%**	86(64)	49(36)	135
**ER+ & (PR<20%, or HER2+ or Ki67 ≥14%)**	17(39)	27(61)	44
**Total**	103	76	179

70-GS 70-gene signature, *Ki-67* proliferation marker protein. The overall concordance between ki67 and the 70-GS was 65%. Kappa 0.20 95% CI 0.048 – 0.35. Using a 14% Ki67 cut off value, 6 patients could not be stratified into Ki67 'low' or 'high' based on their Ki67 percentage and were excluded from the current analysis.
